# The role of the immune system in the pathogenesis of NAFLD and potential therapeutic impacts of mesenchymal stem cell-derived extracellular vesicles

**DOI:** 10.1186/s13287-022-02929-6

**Published:** 2022-06-07

**Authors:** Zahra Moayedfard, Farnaz Sani, Aliakbar Alizadeh, Kamran Bagheri Lankarani, Mohammad Zarei, Negar Azarpira

**Affiliations:** 1 Department of Tissue Engineering and Cell Therapy, School of Advanced Technologies in Medicine, Shiraz University of Medical Sciences, Shiraz, Iran; 2 Hematology and Cell Therapy Department, Faculty of Medical Sciences, Tarbiat Modares University, Tehran, Iran; 3 Health Policy Research Center (HPRC), Shiraz University of Medical Sciences, Shiraz, Iran; 4 Renal Division, Brigham and Woman’s Hospital, Harvard Medical School, Boston, MA USA; 5 John B. Little Center for Radiation Sciences, Harvard T.H. Chan School of Public Health, Boston, MA USA; 6 Transplant Research Center, Shiraz University of Medical Sciences, Khalili Street, P.O. Box: 7193711351, Shiraz, Iran

**Keywords:** Mesenchymal stem cells, Extracellular vesicle, Liver disease, miRNA, NAFLD, Immune response, Inflammation, Exosomes

## Abstract

Non-Alcoholic Fatty Liver Disease (NAFLD) is characterized by intra-hepatocyte triglyceride accumulation and concomitant involvement of the immune system with subsequent histological changes, tissue damage, and clinical findings. There are various molecular pathways involved in the progression of NAFLD including lipotoxicity, endoplasmic reticulum stress, and the immune response. Both innate and adaptive immune systems are involved in the NAFLD pathogenesis, and crosstalk between the immune cells and liver cells participates in its initiation and progression. Among the various treatments for this disease, new cell based therapies have been proposed. Extracellular vesicles (EVs) derived from mesenchymal stem cells (MSC) (MSC-EVs) are new cell-free vehicles with low immunogenicity, which can suppress detrimental immune responses in inflamed tissues. This review aimed to express the immune system’s molecular pathways associated with the initiation and progression of NAFLD. Then, the possible role of MSC-EVs in the treatment of this entity through immune response modulation was discussed. Finally, engineered EVs enhanced by specific therapeutic miRNA were suggested for alleviating the pathological cellular events in liver disease.

## Introduction

Non-alcoholic fatty liver disease (NAFLD) is characterized by intra-hepatocyte triglyceride accumulation and concomitant involvement of the immune system with subsequent histological changes, tissue damage, and clinical findings. The situation varies from isolated excessive hepatocyte triglyceride deposition and simple steatosis (non-alcoholic fatty liver (NAFL) and parenchymal inflammation and hepatocyte injury (non-alcoholic steatohepatitis (NASH) to hepatic fibrosis and cirrhosis and/or hepatocellular carcinoma [1]. Recently, Nan et al. in a systematic review declared several dimensions for changing the name from NAFLD to metabolic dysfunction-associated fatty liver disease (MAFLD) and adoption of a set of positive criteria for disease diagnosis that are independent from alcohol intake and other liver diseases [2].

Obesity, as a major public health issue, is one of the most common risk factors for NAFLD, which is associated with a myriad of comorbidities including type 2 diabetes mellitus, cardiovascular disease, and malignancy [3]. Other risk factors of NAFLD include diabetes mellitus and endocrine diseases such as hypothyroidism, Cushing’s syndrome, growth hormone deficiency, and polycystic ovary syndrome. Patients may also suffer from NAFLD in severe cases of malnutrition like kwashiorkor, short bowel syndrome, and anorexia nervosa as well as after gastroplasty, small bowel resection, jejunum/ileum anastomosis, and drug therapy using tamoxifen, amiodarone, and antiviral or corticosteroids drugs [4, 5].

With respect to pathogenesis, Day and James proposed the ‘two-hit’ theory 20 years ago. Accordingly, hepatic fat accumulation causes a ‘first hit’ (steatosis) that predisposes the liver to a ‘second hit’ of oxidative stress due to increased lipotoxicity and the subsequent necroinflammation, which leads to advanced NASH [6]. The accumulation of such lipids as diacylglycerol increases insulin resistance, mitochondrial and endoplasmic reticulum stress, and autophagic defects, known as lipotoxicity. This event triggers immune responses in Kupffer cells (KCs) and hepatic stellate cells infiltration of neutrophils and lymphocytes, eventually leading to fibrosis and death of hepatocyte cells [7].

NASH is pathologically a complicated condition, which includes a cross talk between many metabolically active tissues (adipose tissue and the liver/gut axis). Hence, several processes may associate with liver injury and inflammation. According to the ‘multiple hits’ theory, multiple insults may take place in parallel including insulin resistance, hepatic lipid accumulation, oxidative stress, mitochondrial dysfunction, gut microbiota, nutritional factors, and genetic and epigenetic factors [8].

Evidence has suggested that exercise may decrease liver fat, even without losing weight. Additionally, different drugs and diets exist for reducing liver fat, but none of them has been fully approved. Recently, mesenchymal stem cells (MSCs) and their extra-cell vesicles (EVs) have been considered for the treatment of various liver diseases [9]. MSCs are often termed ‘conducting cells’ or ‘medical signaling cells’ and can be isolated from many tissues such as bone marrow, umbilical cord, and adipose tissue [9]. It has been elucidated that MSCs can improve inflammatory conditions in liver diseases via secretion of cytokines, chemokines, and growth factors [9]. The secretome of MSCs is released as a heterogeneous population of small membrane-surrounded structures released by different cells in the extracellular environment and bloodstream and is categorized into different groups based on their size, synthesis route (exosomes, ectosomes, and microparticles), and apoptotic bodies. EVs have been considered hypo-immunogenic in a similar fashion to their cells of origin, i.e., MSCs [10]. MSC-derived EVs (MSC-EVs) exert therapeutic functions in kidney, brain, and lung diseases under various preclinical and clinical conditions [11]. However, when excessive fat is deposited in the hepatocytes and metabolic status is changed, the immune microenvironment is altered, as well. Numerous studies indicated that immune cells in the liver played critical roles in the pathogenesis of NAFLD and NASH, but the exact immunological mechanisms leading to liver inflammation and fibrosis have still remained unknown. Among the various treatments for NAFLD, new therapies based on the immunomodulatory effect of EVs have been proposed.

The present review study aims to describe the recent literature on the role of the immune system in the pathogenesis of NAFLD. Briefly, the study aims at summarization of the multiple factors involved in the pathogenesis of NAFLD as well as the cross talk between the immune response and inflammation in the liver and molecular pathways associated with the initiation and progression of NASH. Then, the possible role of MSC-EVs in the treatment of NAFLD through modulation of the immune system will be explained.

## Molecular mechanisms involved in the progression of NAFLD

### NAFLD and lipotoxicity

Accumulation of triglycerides in the liver can be considered a defense mechanism for balancing excess free fatty acids in blood. Therefore, the accumulation of triglycerides is not normally toxic. However, under certain conditions such as inhibition of key enzymes involved in the formation of triglycerides, dysfunction of beta-oxidation fatty acids, and lipolysis of the adipose tissue in insulin resistance, triglycerides accumulation leads to toxic metabolites followed by hepatocyte injury. Toxic lipids result in cellular damage by altering the function and biology of intracellular organelles like the endoplasmic reticulum and mitochondria or by altering the intracellular pathways and interacting with specific pre-inflammatory cell kinases positioned on the cell surface or in the cytoplasm. Eventually, lipotoxicity leads to the development and progression of NAFLD to NASH [12].

### Endoplasmic reticulum stress and NAFLD

Endoplasmic reticulum (ER) is a cellular organelle involved in protein and lipid synthesis in eukaryote cells. The ER stress is an intracellular event that occurs after the accumulation of misfolded or unfolded proteins in order to maintain cell survival. The unfolded protein response (UPR) is responsible for misfolded proteins degradation [13]. When ER hemostasis is disturbed, the UPR pathway is activated by three transmembrane sensor proteins including inositol requiring enzyme 1a (IRE1a), double-stranded RNA-dependent protein kinase (PKR)-like ER kinase (PERK), and activating transcription factor 6 (ATF6) [14]. The UPR is also activated by molecular markers including spliced X-box-binding protein-1 (sXBP1), C/EBP homologous protein (CHOP), and activating transcription factor 4 (ATF4), which ameliorate the stressed state of the ER [15–17]. Prolonged ER stress stimulates the inflammatory response via production of tumor necrosis factor-alpha (TNF-α) and activating nuclear transcription factor-kappa B (NF-κB). On the other hand, the continuous expression of CHOP suppresses the anti-apoptotic factors, eventually resulting in apoptosis. In the liver, ER stress plays a critical role in the progress of steatosis and development of NAFLD [18]. Generally, three main pathways are involved in the development of NAFLD. The hepatic IRE1a/XBP1 and eIF2α/ATF4/FGF21 pathways are important arms for lipid synthesis and very low-density lipoprotein (VLDL) assembly and secretion. Through a negative feedback loop, FGF21 can control the VLDL receptor and ameliorate hepatic fat accumulation [19]. Peroxisome proliferator activated receptor (PPAR) β/δ regulates hepatic VLDLR and triglyceride accumulation in both humans and mice [20–22]. It has been shown that a modest increase in basal liver lipid contents exacerbates pharmacological ER stress-induced liver steatosis through enhancement of lipogenic genes (PPARγ, C/EBPb, and C/EBPd) and reduction in VLDL secretion after the hepatic deficiency of IRE1a [23, 24]. The second pathway of the UPR is PERK/p-eIF2a/ATF4, which is involved in pharmacological ER stress-induced hepatic steatosis, as well [25]. ATF4 plays an important role in regulating FGF21 and VLDLR, which modulate lipid metabolism in the liver. Guozhi Xiao demonstrated that ATF4 depletion reduced hepatic lipogenesis, with small effects on the hepatic VLDL-TG production or fatty acid oxidation. This function prevented excessive lipid accumulation in the liver and prevented the progression of hypertriglyceridemia in fructose-fed ATF4-deficient mice [26]. However, no changes were observed in ATF4 protein levels in mice with fructose feeding [20]. The last arm of UPR is ATF6. Hepatic over-expression of the active form of ATF6 improved hepatic fatty acid oxidation and liver function in diet-induced insulin-resistant mice [27]. CHOP, as another mediator of UPR, induced cell death and inflammatory responses after saturated free fatty acid exposure by activating NF-κβ through a pathway involving IRPK2 expression, leading to the secretion of cytokines like interleukin-8 (IL-8) and TNF-α directly from hepatocytes [18]. ER stress is also related to chronic inflammation via the excessive production of reactive oxygen species (ROS) and activation of NF-κβ and c-Jun N-terminal kinase (JNK) pathways [28]. In a diabetic mouse model, excessive free cholesterol in the liver accumulated in mitochondria and ER led to the enhanced mitochondrial generation of ROS and apoptosis through the JNK1 pathway. These results were dependent on the palmitic acid concentration, to which hepatocytes were exposed [29]. On the other hand, ER stress could increase inflammation through activating numerous inflammatory response pathways in some cellular models and diseases by UPR and downstream pathways including NF-κβ, JNK, ROS, IL-6, and TNF-α [30].

### Inflammasomes and NAFLD

Numerous studies have indicated that toll-like receptor (TLR) signaling can act via the activation of inflammasomes. Inflammasomes are multi-protein complexes located in the cytoplasm that can be activated by both exogenous pathogen-associated molecular patterns like lipopolysaccharide and internal host damage-associated molecular patterns (DAMPs) [31]. DAMPs released by dying hepatocytes can enhance inflammation by activating their respective pattern recognition receptors on KCs and driving the recruitment of inflammatory cells such as monocytes and neutrophils [32]. Similarly, microparticles including the DAMPs mitochondrial DNA derived from steatotic hepatocytes motivate a pro-inflammatory response in KCs/macrophages in a TLR9-dependent manner, as shown in a mouse high-fat diet (HFD) model [33]. Another DAMP released from injured hepatocytes is extracellular adenosine triphosphate [34]. In in vitro studies, adenosine triphosphate contributed to lipopolysaccharide PS-induced IL-6 secretion by mouse KCs. Thus, DAMPs derived from dead hepatocytes might potentiate hepatic inflammation in NASH [35].

The ligand membrane receptors activate a complex intracellular cascade that leads to the secretion of many cytokines including IL-18 and IL-1β, which have pro-inflammatory and profibrotic effects. The involvement of TLR2, 4, and 9 has been shown in the NAFLD/NASH model [36]. The role of TLR2 in the pathogenesis of NAFLD has been disputed, as well. In a dietary model, TLR2 deficiency decreased hepatic steatosis [37]. Likewise, in dietary-induced NASH models, TLR2 and palmitic acid cooperatively activated the inflammasome in KCs. In addition, TLR2-deficient mice revealed diminished inflammasome activation and reduced liver inflammation [36]. Moreover, TLR4-deficient mice showed a significantly lower liver injury and lipid accumulation [38], while TLR9-deficient mice presented decreased hepatic steatosis, inflammation, and fibrosis. These differences were mainly related to the IL-1β produced by KCs in the NASH model [39]. Several reports have demonstrated that TLR4/MyD88 signaling in the liver parenchymal cells played a key role through the primary development of NAFLD [40]. Moreover, bone marrow-derived or non-bone marrow-derived cells participated in inflammasome activation in a TLR9/MyD88-dependent pathway in the steatohepatitis model [41]. Inflammasome-derived inflammatory cytokines also played a major role in the progression of steatosis to steatohepatitis and liver fibrosis in hypercholesterolemic mice [42]. IL-1β, IL-1α, and IL-33 can start the cascade of changes leading to steatohepatitis. It has been indicated that IL-1β can motivate the secretion of TNF and IL-6 and enhance the accumulation of triglycerides and cholesterol by upregulation of the genes containing diacylglycerol-O-acyltransferase and PPARg that are involved in their synthesis. Different studies have revealed that IL-33 administration recovered steatosis, but enhanced some ingredients of NASH including fibrosis. Besides, patients with fibrosis had increased transcript levels of IL-33 in the liver [43–45].

### Innate immune system in NAFLD

The innate immune system is involved in NASH by activating the resident KCs through recruitment of immune cells including neutrophils, monocytes, and natural killer (NK) and natural killer T (NKT) cells to the liver. These cells are also related to inflammation by producing cytokines, chemokines, eicosanoids, nitric oxide, and ROS [8, 46].

Many pro-inflammatory functions are involved in NAFLD progress [46]. For instance, KCs are directly stimulated by excess free fatty acids and cholesterol. Moreover, the release of chemokines (CC-chemokine ligand 1 (CCL1), CCL2, and CCL5) differentiates monocytes into M1 activated macrophages [47]. In a phase II clinical trial, blocking the CCL2 and CCL5 signaling alleviated NASH and diminished fibrosis evolution [48]. Blocking anti-inflammatory agents controlling macrophage responses also declined the progression of NASH under different circumstances [49].

#### Macrophages in NAFLD

Liver macrophages contain subsets of liver cell populations. KCs include a population of macrophages inside the liver originating from yolk sac-derived erythromyeloid progenitors in the fetal liver. They locally proliferate and self-sustain [50, 51]. Penetrating monocytes are obtained from circulating monocytes expanded from bone marrow-resident hematopoietic stem cells [52].

In addition to liver resident KCs, a large population of macrophages was recruited into liver in NAFLD and NASH. These infiltrated macrophages can be distinguished from KCs by the expression of several surface markers. Macrophages have an essential role in the immune homeostasis and the development of other liver diseases that share main endpoints with NAFLD including chronic viral hepatitis and alcoholic liver disease. KCs are activated as the first responder in contact with immunological materials in steatosis. The transition from steatosis (mild form of NAFLD) to NASH depends on the development of liver inflammation. KCs are also motivated in the beginning of steatohepatitis through TLR signaling. TLRs are the beginning agents of inflammation in a liver with steatosis. TLR9 activates the release the KCs of IL-1β, which is implicated in hepatocyte lipid accumulation, fibrogenesis, and cell death [39]. Balance among macrophage polarization states is effective in the development of steatohepatitis. Anti-inflammatory macrophages (M2) can induce their M1 KCs apoptosis, which has been reported to diminish the progression of fatty liver diseases [53]. Furthermore, the advantageous effects of IL-10 macrophages included the motivation of lipid catabolism followed by the prevention of hepatocytes inflammation [54].

In reparative phases of acute inflammation as in NASH, the liver macrophages can undergo activation to M2-type macrophages, which have an immunosuppressive but pro-fibrogenic phenotype. The activated macrophages can produce high levels of transforming growth factor-β1 and IL-13, causing progressive fibrosis [55]. It has been reported that KCs can secrete and respond to pro-inflammatory cytokines like IL-6 as well as to anti-inflammatory cytokines such as IL-10. The role of macrophages has been mentioned in a variety of studies on NAFLD. For instance, a study conducted among Korean young adults demonstrated a large number of CD68 + KCs in biopsy samples of patients with progressive NAFLD [56]. In another study, increased activated macrophages were found in the spaces among injured hepatocytes in children with NAFLD [57]. Portal influx of macrophages also sounds to be the onset of NAFLD in humans, happening at the steatosis stage before advanced inflammation or fibrosis, but predicting a more severe disease [58]. Furthermore, hepatic crown-like structures were detected around steatotic hepatocytes, which included lipogranuloma and macrophage alignment [59, 60].

Infiltration of F4/80+ macrophages and activation of KCs with enhanced expression of pro-inflammatory cytokines were observed in the lipotoxic high-fat and high-cholesterol diet in a NASH model [61]. In the same line, hepatic macrophage infiltration was found in different models of mouse dietary such as methionine–choline-deficient diet and high-fat diet, which was related to increase aging among mice [62–64].

#### Neutrophils

Infiltration of neutrophils occurs in both patients and mice with NASH. Recruitment of neutrophils into the liver occurs by few chemokines including CXCL1, IL-8, and CXCL2 in the very early stage of liver injury in NAFLD [65]. Many studies have shown that the release of neutrophil-specific components is involved in the progression of NASH. For example, a report indicated that suppression of elastase in the early stage alleviated the severity of NASH in a mouse model [66]. In the same line, Zang et al. showed that the ratio of the serum neutrophil elastase to its endogenous inhibitor a1-antitrypsin was associated with the degree of liver inflammation in patients with NASH [67]. Mirea et al. also suggested that neutrophil proteinase-3 and elastase concentrations increased, while the level of a1-antitrypsin was decreased in obese patients compared to healthy controls. Additionally, proteinase-3 and elastase levels were closely associated with liver fibrosis in NASH [68]. Another study revealed an increase in an important neutrophil-derived enzyme, i.e., myeloperoxidase (MPO), in NASH patients, which might contribute to liver inflammation and development of NASH [59]. Moreover, neutrophils and MPO from neutrophils might activate hepatic stellate cells (HSCs) and induce liver fibrosis in NASH [69]. Nevertheless, it is not very clear how neutrophils contribute to the development of NAFLD.

#### Natural killer T cells

NKT cells are the key ingredients of hepatic lymphocytes that express both NK cell markers and T cell markers. They are stimulated by lipid and glycolipid antigens presented by CD1d that can be expressed by hepatocytes, macrophages, dendritic cells (DCs), and B cells. NKT cells are activated by IL-12, which is released by DCs and KCs, resulting in Fas-mediated target cell lysis. NKT cells release cytokines like IL-4 and IFNγ. In addition to classical cytokines, NTK can secrete OPN, which is implicated in liver injury progression [70]. NKT cells also display immunogenic functions during acute liver inflammation [71]. NKT cells are generally depleted in liver steatosis, but are increased in NASH-related liver fibrosis [72]. In the early stage of NAFLD, NKT cells are reduced and skewed to a pro-inflammatory Th1 cytokine profile. However, in the advanced stage of NAFLD such as NASH, NKT cells are increased in the liver and contribute to the development of liver fibrosis [73]. In the research carried out by Adler et al., NKT cells significantly increased in the liver and blood of patients with moderate-to-severe steatosis, which supported the role of NKT cells in NAFLD [74].

A previous study demonstrated that liver inflammation induced by the administration of α-galactosyl ceramide in mice led to IL-17 secretion by NKT cells. Besides, administration of IL-17 neutralizing monoclonal antibodies before α-GalCer injection significantly exacerbated hepatitis and increased the infiltration of neutrophils and monocytes, upregulation of CCL2 and CXCL5, and progressive inflammation in the liver. In contrast, prescription of exogenous recombinant murine IL-17 before α-GalCer injection ameliorated hepatitis and inhibited the recruitment of liver inflammatory monocytes [75].

It has been reported that liver cells constitutively express low levels of NKG2D cytotoxic mediators (like MIC-A and MIC-B), which are increased under stress conditions including viral infection or NASH [76].

#### Natural killer cells

NK cells conduct cytotoxic attacks against target cells by perforin-mediated mechanisms or cell–cell interaction (e.g., Fas–Fas ligand), as a key component of the innate immune system in the liver kill pathogens, tumor cells, stressed hepatocytes, and hepatic stellate cells (HSCs). NK cells can also act as regulatory cells that influence DCs, KCs, T cells, B cells, and endothelial cells by releasing various cytokines (such as IFN-γ, TNF-α, and IL-10), chemokines, and growth factors or through innate immune recognition.

Kahraman et al. disclosed that the expression of several NK cells associated with cytotoxic ligands (like TNF-related apoptosis-inducing ligand (TRAIL), NKG2D, and MICA/B mRNAs) was significantly increased in obese patients with NASH. They also reported that the expression of MICA/B mRNAs was positively correlated with NAFLD activity and hepatocyte apoptosis in patients with NASH [77]. Similarly, another study indicated that many NK cell ligands were upregulated in the livers of mice with NASH, which was followed by the infiltration of activated cytotoxic NK cells [78]. In addition, NK cells could promote the pro-inflammatory condition in the steatotic liver by TRAIL secretion, thereby contributing to development toward steatohepatitis [79]. Furthermore, IL-15 stimulated NK cell activation and promoted NASH development in mice. NK cells also played a crucial role in regulating progressive fibrosis in NASH [80]. Yet, further studies are required to clarify the roles of NK cells during NASH.

#### Dendritic cells

Dendritic cells (DCs) originate from hematopoietic progenitor on bone marrow cells. Liver DCs include a heterogeneous population of sinusoidal antigen-presenting cells in the liver located preferentially in the pericentral and periportal space C, which comprise less than 1% of non-parenchymal cells [81]. Lately, DCs have been mentioned as the mediator of non-infectious chronic inflammatory conditions and the powerful inducer of immune response. These antigen processing cells induce T-cell-mediated immunity. The function of DCs includes their capability to capture antigens and their ability to process and represent peptides and transmit them to lymphoid organs [82]. Heier et al. distinguished CD103‏ classic type I DCs as a protective DC subtype that influenced the pro-anti-inflammatory balance, protected the liver from metabolic damage, and played a key role in the inflammatory process during the development of steatohepatitis in mice. In the mice lacking CD103‏ DCs, the progression of NASH was enhanced, while the adoptive transfer of CD103‏ DCs ameliorated inflammation and steatosis in the liver, suggesting the protective role of CD103‏ DCs in NASH [83].

In an MCD diet in the early phase of NASH progression, DC maturation occurred in the form of the expression of major histocompatibility complex class II (MHC II) and CD40 that is essential for antigen presentation as well as the expression of co-stimulatory agents CD80, CD54, and CD86 that are necessary for activation. In that study, analysis of several DC populations indicated a decrease in plasmocytoid DCs (B220 +) deficiency, an increase in CD11b + CD8a − myeloid deficiency, and a decline in the fraction of CD11b − CD8a + lymphoid DCs [84].

Ibrahim et al. found two DC populations in both mice and humans specified by different concentrations of high (high-DC) and low (low-DC) lipid contents and expression of markers in the liver. In addition, different lipid profiles in DCs were related to the lipid-rich microenvironment in the liver. Moreover, a higher percentage of high-DC fraction was observed in steatohepatitic livers in both human and animal models [85].

The liver DC population with high lipid concentrations was found to be immunogenic. High-DCs stimulated NK and NKT cells and increased the secretion of different pro-inflammatory cytokines and chemokines, while low-DCs were virtually non-productive and induced regulatory T cells, anergy to cancer, and oral tolerance in liver. The main cytokines of high-DCs were TNF-α, IFN-γ, IL-2, IL-4, and IL-6 [85]. Another study showed an increase in the secretion of pro-inflammatory cytokines in hepatic DCs in NASH. The results also revealed the in vitro stimulation of the proliferation of antigen-restricted CD4 + T cells and allogeneic T cells as well as the downregulation of Tregs expression [86]. Furthermore, Henning et al. emphasized that DCs restricted CD8 + T cell proliferation and limited TLR expression and cytokine secretion in the innate immune effector cells such as neutrophils, KCs, and inflammatory monocytes in NASH. Despite their regulatory task in NASH, ablation of DC populations during the disease recovery phase led to the delayed resolution of inflammation and fibroplasia in the liver. DCs could also induce NK and T cells and produce high amounts of TNF in liver fibrosis [86].

### Adaptive immunity

Adaptive immunity has been described by antigenic specificity, immunologic memory, diversity, and self-non-self-recognition. This immune response includes B and T lymphocytes and a diversity of molecules that coordinate cellular interactions [87].

#### T cells

The role of conventional T cells in the pathogenesis of NASH and NAFLD has been well distinguished. In an NAFLD model, the MCD diet resulted in a reduction in the number of intrahepatic CD4‏ T cells but not CD8‏ T cells as well as an increase in the production of IFN-γ, IL-17, and ROS in intrahepatic T cells [88]. In the same line, increase in T cell-derived Th-17 cells was confirmed in NAFLD and NASH. Hence, the regulation of the Th-17-associated genes (ROR-γt, IL-17, IL21, and IL23) was confirmed in patients with NASH [89]. Additionally, Tang et al. found that the neutralization of IL-17 in the mice fed by HFD reduced the lipopolysaccharide-induced liver damage, as demonstrated by reduced alanine aminotransferase level and inflammatory status. Moreover, IL-17 together with free fatty acids contributed to the progression of steatosis via insulin signaling pathway interference [89]. Nonetheless, neutralization or IL-17RA deficiency with anti-IL-17mAb protected mice from diet-induced liver injury and liver steatosis and suppressed KCs activation. Thus, IL-17 was suggested to be associated with the development of NAFLD in the mouse model. Evidence has also confirmed that IL-17 was directly involved in the fibrosis of liver by activating the STAT3 pathway in hepatic stellate cells (HSCs) [90].

Although IL-17-generating T cells were increased, regulatory T cells (Tregs, the negative regulators of inflammation) were reduced in fatty liver disease. Low levels of hepatic Tregs in the high-fat diet model led to the suppression of inflammatory responses [91]. Interestingly, KCs were interrelated with antigen-specific T cells and increased IL-10-expressing Treg cells in a mouse model [92]. In a steatotic model, an increase was observed in the number of Th1 cells and the Th1-related cytokines, i.e., IL-12, IFNγ, and TNFα, during the progression of NAFLD/NASH [93].

#### B cells

B cells are specific cells generating antibodies in the adaptive immune system. B lymphocytes create nearly 6% of intrahepatic cells [94] and produce the B cell activating factor (BAFF), which regulates the process of B cells maturation [95].

Up to now, few studies have been conducted on the role of B cells in NAFLD pathogenesis. Zhang et al. demonstrated that intra-hepatic B-lymphocyte cells contributed to NAFLD not only through the secretion of cytokines and immunoglobulin, but also through the regulation of intrahepatic CD4^+^ intra-hepatic T-lymphocyte cells in the mice fed with HFD [96]. The results of another study revealed that BAFF receptor-deficient mice showed an improvement in HFD-induced obesity and insulin resistance accompanied by a decrease in the number of B cells and serum IgG level. In an animal study, blocking BAFF signaling enhanced insulin resistance in HFD-fed mice, but promoted liver steatosis via activating the liver fat metabolism [97]. Barrow et al. found that B cell deficiency improved NASH development, and adoptive movement of B cells from NASH livers recapitulated the disease. Mechanistically, activation of B cells through NASH involved signaling through the innate adaptor, i.e., myeloid differentiation early response protein 88 (MyD88). B cell-specific elimination of MyD88 reduced hepatic T cell-mediated inflammation and fibrosis, but had no effects on steatosis [98]. These findings confirmed that B cells maintained metabolic homeostasis. Hence, manipulation of these cells has been suggested as a potential therapy for NAFLD.

### MSC and EV

MSCs are self-renewing, multipotent progenitors, which can be isolated from several sources including bone marrow, Wharton’s Jelly, adipose tissue, umbilical cord blood, and placenta [99]. They have been extensively examined in clinical studies due to their multiple biological roles such as multi-lineage differentiation, tissue repair elevation, and neuro-protection as well as their anti-inflammatory and immunosuppressive properties [100]. It was thought that MSCs located in damaged tissues could differentiate and substitute injured cells. However, a recent study indicated little and transient MSCs engraftment at injury sites [101]. Therefore, it has been suggested that MSCs show their therapeutic functions mostly through the secretion of trophic elements. Thus, the effective functions of MSCs can be determined from the secreted factors rather than from their tissue differentiation and intercalation. Some studies have proved that the MSCs secretome led to organ healing by inducing a move from pro-inflammatory to anti-inflammatory cytokine secretion in the site of injury [102]. In this context, scientists have focused on the characterization of MSCs-secreted soluble agents (i.e., cytokines, chemokines, and growth factors), and endosome-derived exosomes have been considered as physiologically strong ingredients of the MSCs secretome [103]. Similar to soluble agents, EVs are a principal instrument in cell–cell communication. There are several subtypes of EV, among which exosomes are the paracrine effectors of MSCs and the regulators of cell–cell communication [104]. Exosomes are approximately 40–150 nm extracellular vesicles, which are specified by the expression of specific markers like CD81, CD63, and CD9 [105].

MSCs are viable cells with both self-renewal and differentiation capacities. Their conventional clinical usage raises a few critical concerns: (1) loss of stemness induced by time/aging, (2) undesired differentiation of MSCs and its dire consequences like tumorigenesis, calcification, and ossification, (3) development of malignancies due to the transfer of cells with mutated or damaged genetic materials, and (4) perpetuation of MSCs in the body after disease resolution due to the prolonged viability of the cells. Conversely, EV-based MSC therapies are not associated with the mentioned concerns and risks. The therapeutic properties of EVs also appear to transcend those of other biological or synthetic nanoparticles. For instance, EVs display longer circulating half-lives and are more biocompatible compared to liposomes and polymeric nanoparticles [106]. Thus, EVs have similar functions as their cells of origin and since they do not carry the risk of cell transplantation such as tumor formation or small vessel blockage, they can be considered a potential therapeutic tool for cell-free therapy [107].

### The immunomodulatory role of MSC-derived exosomes as a treatment option (in vitro)

Many studies have revealed the immunomodulatory effects of MSC-EVs in different diseases. Blazquez et al. showed that MSC-derived exosomes exerted an inhibitory effect on the differentiation and activation of T cells and decreased T cell proliferation and IFN-γ release [108].

Del Fattore et al. recently reported that the MSCs immunosuppressive effects on the B lymphocytes in peripheral blood mononuclear cell (PBMC) culture could be reproduced by the EVs isolated from the culture supernatants of MSCs. In that study, MSC-EVs were shown as the conveyors of the strong dose-dependent inhibition of B cell proliferation and differentiation in a CpG-stimulated PBMC system [109]. They also investigated the effect of MSC-EVs and MSC-EVs on T cells on PBMC cultures stimulated by anti-CD3/CD28 beads. The findings indicated that co-culture with MSCs inhibited the proliferation of activated T cells, without causing significant changes in apoptosis. Additionally, MSC-EV/PBMC co-cultures exerted a quite different effect, since they induced T cell apoptosis without significantly suppressing cell proliferation and Treg proliferation. These findings revealed a net immunosuppressive effect, which was confirmed by the increased concentration of the anti-inflammatory cytokine IL-10 in the culture medium [110].

Mokarizadeh et al. reported that murine BM-MSCs activated by IL-1β induced T cells apoptosis, secretion of IL-10 and TGF-β, and production of Tregs in murine auto-reactive lymphocytes. They also demonstrated that MSCs-derived micro-vesicles served as vehicles for MSCs-specific tolerogenic molecules including PD-L1, Gal-1, and TGF-β. These results suggested MSC-EVs as effective agents inducing peripheral tolerance and modulating immune responses. Hence, indirect MSCs application has been recommended for the treatment of autoimmune diseases [111].

Favaro et al. found that human bone marrow MSC exosomes induced the downregulation of Th1 responses, enhanced the Treg proportion, and decreased the percentage of Th-17 cells in PBMCs from patients with type I diabetes [112].

### The immunomodulatory role of MSC-derived exosomes as a treatment option (in vivo)

In various preclinical models (Table 1), the immunomodulatory effect of MSC-secreted exosomes improved the therapy-refractory graft-versus-host disease (GVHD) and promoted organ healing [113–116]. In a mouse model of GVHD, co-incubation of MSC-derived EVs by THP-1 cells induced the creation of CD4 + CD25 + T cells or CD4 + CD25 + Foxp3 + Tregs from CD4 + T cells activated by allogeneic APC-enriched CD11C + cells. In that study, MSC exosome reduced GVHD symptoms and enhanced survival [113]. Fujii et al. showed that CD4 + and CD8 + T cells were suppressed in EV-treated GVHD mice [114]. Importantly, the ratio of CD62L-CD44 + to CD62L + CD44-T cells was reduced, suggesting that BM-MSC-derived EVs suppressed the functional differentiation of T cells from a naive to an effector phenotype [114].

**Table 1:** Overview of an application of MSC-EVs in experimental studies

Cell source	Experimental model	Administration rout	Result	References
Bone marrow	Dilated cardiomyopathy	Intravenous	Reduction in the expression levels of IL-1, IL-6, and TNF-α Reduction in circulating macrophages Promotion of the conversion of macrophages from pro-inflammatory to anti-inflammatory status	Sun et al. [118]
Umbilical cord blood	Myocardial ischemia–reperfusion	Intracardiac	Immune-suppressing effect of miRNA-181a exosomes	Wei et al. [119]
Bone marrow	Traumatic-brain injury	Intravenous	Reduction in neuroinflammation	Zhang et al. [120]
Bone marrow	Post-ischemic neurological impairment	Intravenous	Attenuation of post-ischemic immunosuppression in the peripheral blood	Doeppner et al. [121]
Bone marrow	Focal brain injury	Intra-arterial	Infiltrating leucocytes including T cytotoxic cells Significant decrease in pro-inflammatory cytokines and chemokines	Dabrowska et al. [121]
Wharton’s Jelly	Renal ischemia–reperfusion injury	Intravenous	Alleviation of inflammation suppression of the expression of chemokines Decrease in the number of macrophages in the kidney	Zou et al. [123]
Bone marrow	Acute lung injury	Intratracheal	Reduction in inflammation	Zhu et al. [124]
Bone marrow	Acute lung injury	Intratracheal	Reduction in pro-inflammatory cytokines	Khatri et al. [124]
Bone marrow	Pulmonary fibrosis	Intravenous, intracardiac	Increase in an immunoregulatory, anti-inflammatory monocyte phenotype	Mansouri et al. [126]
Bone marrow	Acute respiratory distress syndrome	Intranasal	Reduction in inflammation	Morrison et al. [127]
Bone marrow	Colitis	Intravenous	Downregulation of pro-inflammatory cytokines	Yang et al. [128]
Umbilical cord	Inflammatory bowel disease	Intravenous	Increase in IL-10 Reduction in pro-inflammatory cytokines Decrease in the infiltration of macrophages into the colon tissues	Mao et al. [129]
Intravenous	Sepsis syndrome	Intravenous	Suppression of the inflammatory reactions by healthy exosomes	Chang et al. [131]
Bone marrow	Graft-versus-host disease	Intravenous	Reduction in activation and infiltration of CD4 + T cells Inhibition of IL-17-T cells Induction of IL-10 regulatory cells Reduction in pro-inflammatory cytokines	Lai et al. [115]
Umbilical cord	Graft-versus-host disease	Intravenous	Lower absolute numbers of CD3 + CD8 + T cells Reduction in the serum levels of pro-inflammatory cytokines A higher ratio of CD3 + CD4 + and CD3 + CD8 + T cells Higher serum levels of IL-10	Wang et al. [116]
Umbilical cord	Autoimmune uveitis	Periocular	Reducing the infiltration of T cell subsets and other inflammatory cells into the eyes	Bai et al. [134]
Adipose tissue	Autoimmune diabetes	Intraperitoneal	Increase in the levels of anti-inflammatory cytokines Decrease in the levels of pro-inflammatory cytokines Increase in the T regulatory cell ratio	Nojehdehi et al. [136]
Bone marrow	Myocardial I/R injury	Intra-myocardial	Reduction in the inflammation level	Zhao et al. [135]
Bone marrow	Rheumatoid arthritis	Intravenous	Delaying inflammation	Zheng et al. [133]
Embryonic stem cell	Graft-versus-host disease	Intravenous	Increased Treg production in vitro and in vivo through an APC-mediated pathway	Zhang et al. [113]
Bone marrow	Graft-versus-host disease	Intravenous	Decrease in the ratio of CD62L-CD44 + to CD62L + CD44- T cells Suppression of CD4 + and CD8 + T cells	Fujii et al. [114]

Various studies have reported that the administration of MSC-EVs is superior over cell-based therapy, because it removes the safety concerns associated with the use of most stem cells for patients [117]. In one study, MSC exosomes could remarkably alleviate inflammatory cardiomyopathy by improving the inflammatory microenvironment of myocardium and attenuating the pro-inflammatory macrophages on the JAK2-STAT6 pathway [118].

In a mouse model of myocardial I/R injury, exosomal miRNA-181a could suppress the inflammatory response and increase the Treg cell ratio through inhibition of the c-Fos protein [119]. Zhang et al. also indicated that MSC-generated exosomes could improve functional recovery and reduce neuro-inflammation in rats after traumatic brain injury [120]. Moreover, MSC-EVs could promote post-ischemic neurological recovery by attenuating post-ischemic immunosuppression (including B cell, NKC, and T cell lymphopenia) [121]. In addition, intra-arterial hBM-MSCs transplantation significantly decreased pro-inflammatory cytokines and chemokines compared to non-treated rats with focal brain injury [122].

Zou et al. found that the administration of MVs immediately after renal ischemia–reperfusion could suppress the expression of CX3CL1 and decrease the number of CD68 + macrophages in the kidney [123]. It was also determined that MSCs were effective in reducing inflammation in E. coli endotoxin-induced acute liver injury in C57BL/6 mice [124].

In an animal model, the intratracheal administration of MSC-EVs significantly reduced virus shedding in influenza virus replication as well as the virus-induced production of pro-inflammatory cytokines in the lungs [125].

Mansouri et al. emphasized that mesenchymal stromal cell exosomes prevented and reverted experimental pulmonary fibrosis through the modulation of monocyte phenotypes to an immunoregulatory, anti-inflammatory monocyte phenotype [126]. In another study, MSC-EVs modulated macrophages in clinically relevant lung injury models [127]. Yang et al. suggested that the beneficial effects of BMSC-EVs were due to the reduction in pro-inflammatory cytokines levels and suppression of NF-κBp65 signal transduction pathways in experimental colitis [128]. Similarly, Mao et al. found that exosomes from hucMSCs had profound immunomodulatory effects on alleviating inflammatory bowel disease [129].

Li et al. found that miR-181c expression in MSC-exosomes decreased burn-induced inflammation by downregulating the TLR4 signaling pathway in macrophages [130]. Furthermore, adipose-derived MSCs-derived exosomes alleviated overwhelming systemic inflammatory reaction in a rat model of sepsis [131].

In vivo, human umbilical cord MSCs exosomes repaired damaged liver tissue and decreased the expression of the NLRP3 inflammasome in a mouse ALF model [132].

Zheng et al. showed that the bone marrow-MSCs-secreted exosomal miR-192-5p could delay the inflammatory response in rheumatoid arthritis and might represent a possible therapeutic strategy for the treatment of rheumatoid arthritis [133]. Bai et al. suggested a potential novel therapy for experimental autoimmune uveitis, in which MSC exosomes effectively inhibited the migration of inflammatory cells [134].

It was shown that the administration of MSC-EVs to mice through intramyocardial injection decreased TLR4, NF-kβ, and MyD88 levels in macrophages via miR-182-5p and induced polarization toward an M2 anti-inflammatory phenotype [135]. Nojehdehi et al. found the immunomodulatory effects of MSCs-derived exosomes on experimental type I autoimmune diabetes through increasing the regulatory T cell population and their products without a change in the proliferation index of lymphocytes [136].

Overall, extracellular vesicles, due to their different nature, have different effects on target tissues. That is why a standard separation method is needed for their homogenization. For example, a prior study showed that excessive fatty acid accumulation was toxic to hepatocytes and, consequently, the release of EVs (hepatocyte EVs) was induced via lipotoxicity, which could mediate the progression of fibrosis via the activation of nearby macrophages and hepatic stellate cells [137].

### The immunomodulatory role of MSC-derived exosomes in liver injury

Dorairaj et al. emphasized that excessive fatty acid accumulation was toxic to hepatocytes, and this lipotoxicity could induce the release of EVs (hepatocyte EVs) that could mediate the progression of fibrosis via the activation of nearby macrophages and hepatic stellate cells [137]. They also concluded that adipocyte and hepatocyte EVs were effective in the development of NAFLD and progression of liver injury [137]. Several study has been considered about.

MSC-EVs suppressed the proliferation of PBMCs, enhanced the secretion of anti-inflammatory mediators (i.e., TGF-β and IL-10), reduced IFN-γ, and modulated pro-inflammatory cytokines (TNFα and IL-2) in a chronic liver injury model [138].

The immunosuppressive effects of MSCs-derived exosomes have been associated with a decrease in the number of recruiting macrophages and neutrophils as well as a reduction in TNF-α and IL-6 levels in hepatic injury [139]. In an autoimmune hepatitis experimental model, bone marrow stem cells-derived exosomes prevented liver injury via a mechanism associated with exosomal miR-223 regulation of NLRP3 and caspase-1 [140].

MSC-EVs protected the liver from hypoxia-induced injury or from ischemia–reperfusion harm. The mice that received the injection of MSC-derived EVs before the ischemia revealed hepatocyte proliferation demonstrated by an increased number of Ki-67-positive cells and decreased expression of inflammation-associated genes [141]. Similarly, the immunosuppressive effect of MSC-EVs on liver injury in an animal model was reported by Tamura et al. The results revealed a decrease in the expression of mRNA for pro-inflammatory cytokines IL-1, IL-2, and TNF-α, IFN-γ, but an increase in anti-inflammatory cytokines TGF-b and HGF as well as in the number of T regulatory cells in the liver tissue [142].

Jia et al. showed that the EVs derived from human umbilical cord MSCs relieved rat hepatic ischemia–reperfusion injury by suppressing oxidative stress and neutrophil inflammatory response [143].

**Fig. 1:**
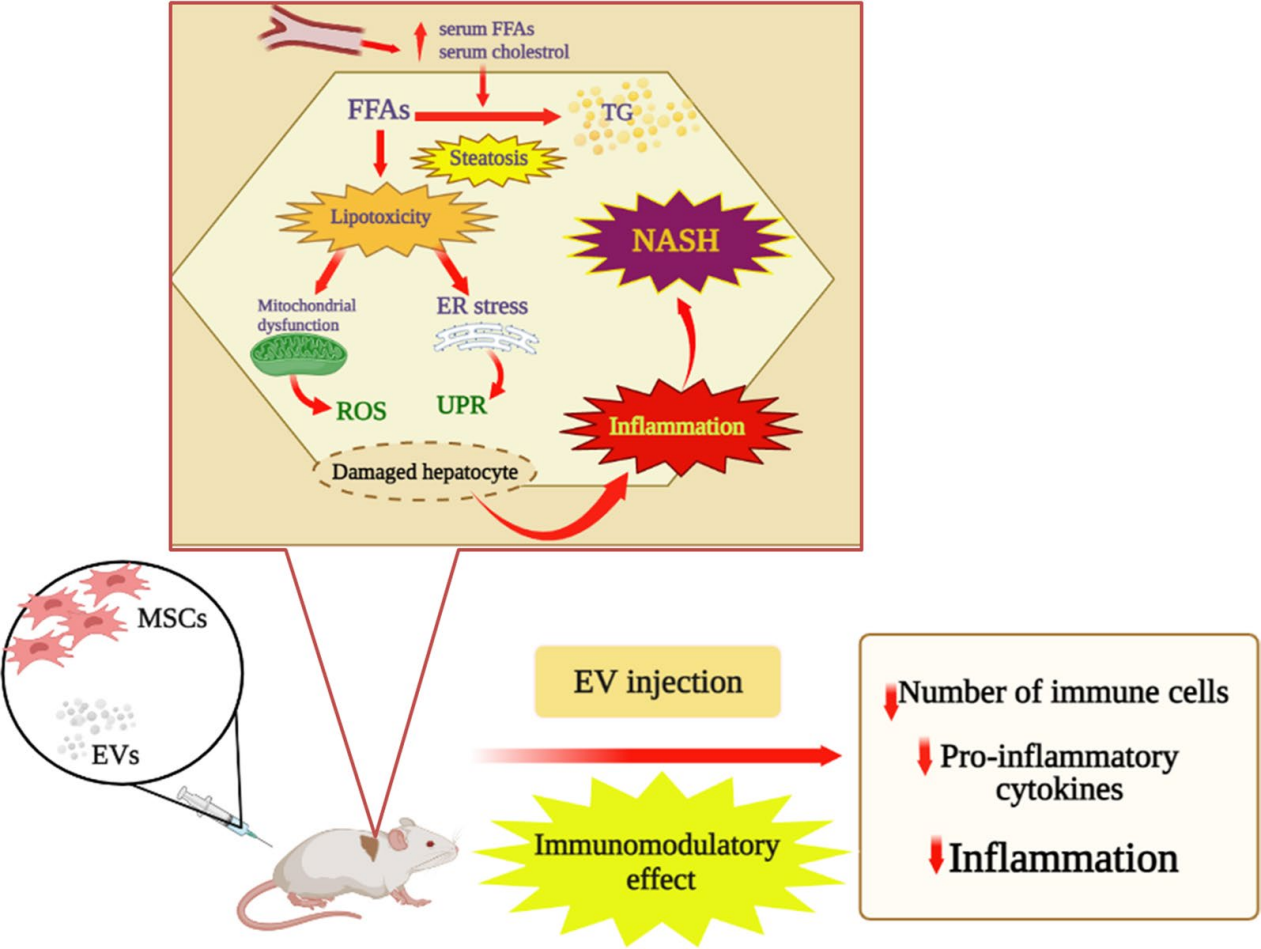
Immunomodulatory effect of MSC-EV in liver injury. FFAs, free fatty acids; TG, triglyceride; ROS, reactive oxygen species; ER, endoplasmic reticulum; UPR, unfolded protein response; NASH, non-alcoholic steatohepatitis; MSC, mesenchymal stem cell; EV, extracellular vesicle. The increased hepatic FFAs flux which derives from the multiple hits leads to two different situations: synthesis and accumulation of triglycerides (TG) (steatosis) and ‘toxic’ levels of fatty acids, free cholesterol, and other lipid metabolites (lipotoxicity) which cause mitochondrial dysfunction with oxidative stress and production of ROS and endoplasmic reticulum (ER) stress with activation of UPR, all leading to hepatic inflammation. In vivo study, immunomodulatory effect of MSC-EVs alleviated inflammation and decreased the number of immune cells and pro-inflammatory cytokines.

Engineered MSCs have been used in preclinical studies. In a research carried out by Lu et al. [144], mouse MSC-EVs carrying miR-223-3p into macrophages effectively attenuated the inflammatory responses in autoimmune hepatitis liver. Additionally, inflammatory cytokines were released in both the liver and macrophages. Moreover, Liu et al. [145] showed that MSC-EV-shuttled miR-17 played an important role in the treatment of acute liver failure by suppressing the secretion of pro-inflammatory mediators, activating NLRP3 inflammasome, and targeting TXNIP in hepatic macrophages. Furthermore, some in vivo investigations revealed that MSC-derived EVs alleviated hepatic injury by modulating the inflammatory response in different models [146]. Ohara and Ohnishi disclosed that EVs derived from adipose MSCs significantly reduced the number of KCs in the livers of the rats with NASH and diminished the mRNA expression levels of inflammatory cytokines such as TNF-α, IL-1β, IL-6, and TGF-β. In addition, adipose MSC-EVs significantly reduced the number of KCs and HSC activation in the rats with liver fibrosis and suppressed HSC and KC activation [147]. Administration of both human MSCs and their EVs decreased inflammatory indicators according to the qualitative real-time polymerase chain reaction (qRT-PCR), improved fibrosis, and increased the liver anti-inflammatory macrophages in a NASH model. This suggested the anti-inflammatory and anti-fibrotic effects of MSCs and their EVs, as an ideal therapeutic method [148]. According to the findings mentioned above, in general, they show the immunomodulatory effect of extracellular vesicles, which reduces the number of immune cells and cytokines produced and ultimately leads to a reduction in inflammation in NASH (Fig. 1). Yet, data obtained from in vitro and animal studies (Table 2), research on non-human primates, and clinical trials are needed to evaluate the safety and efficacy of MSCs-based therapy for NAFLD.

**Table 2:** Overview of an application of MSC-EVs in liver injury

Cell source	Experimental model	Administration rout	Result	References
Bone marrow	Hepatic ischemia Reperfusion injury	Intravenous	Repression of the transcription of inflammation-associated genes	Anger et al. [141]
Bone marrow	Liver injury	Intravenous	Enhancement of anti-inflammatory cytokines and T regulatory cells	Tamura et al. [142]
Bone marrow	Hepatic ischemia Reperfusion injury	Intravenous	A decrease in the number of recruiting macrophages and neutrophils as well as a reduction in TNF-α and IL-6 levels	Haga et al. [149]
Umbilical cord	Acute liver injury	Intravenous	Reduced expression of pro-inflammatory cytokines TNF-α, IL-1β, IL-6, and TGF-β and number of Kuppfer cells	Ohara et al. [147]
Umbilical cord	Hepatic ischemia Reperfusion injury	Intravenous	Inhibition of neutrophil inflammatory response	Yao et al. [143]
Adipose tissue	Acute liver injury	Intravenous	Reduction in inflammatory activation in Kuppfer cells	Liu et al. [145]
Umbilical cord	Acute liver injury	Intravenous	Reduction in NLRP3, Casp-1, IL-1, and IL-6 expressions in the macrophage	Jiang et al. [132]
Embryonic stem cell	Chronic liver injury	Intravenous	Upregulation of anti-inflammatory cytokines (TGF-β1 and IL-10) and downregulation of pro-inflammatory cytokines (TNFα and IL-2)	Mardpour et al. [148]
Bone marrow	Experimental autoimmune epatitis	Intraperitoneal	Regulation of NLRP3 and caspase-1	Chen et al. [140]
Bone marrow	Autoimmune hepatitis	Intraperitoneal	Reduction in inflammatory responses	Lu et al. [144]
Liver tissue	Liver fibrosis	Intravenous	Modulating the inflammatory response	Bruno et al. [146]
Adipose tissue	NASH model	Intravenous	Increase in anti-inflammatory macrophages in the liver	Watanabe et al. [148]

## Conclusion

In summary, immune cells, both innate and adaptive are skewed toward a pro-inflammatory phenotype in livers with NAFLD and NASH and played critical roles in the progression of NAFLD and NASH. Targeting immune cells in the liver may represent one of the effective ways for NASH treatment.

EVs could alleviate inflammation in liver diseases. Therefore, they have been emerged as a therapeutic tool in treating NAFLD due to their ability in stable delivery of drugs, miRNA, siRNA, and other cargoes as well as their easy uptake by liver cells, which paves the way for EV-based therapy. However, further in vitro and in vivo studies are required for better understanding cross talk between EVs and immune cells with clarifying the potency and the mechanisms of action of this novel potential therapeutic tool.

## Data Availability

Not applicable.

## Abbreviations

NAFLD: Non-alcoholic fatty liver disease
MSC: Mesenchymal stem cell
EV: Extracellular vesicles
MSC-EVs: Extracellular vesicles (EVs) derived from mesenchymal stem cells
NASH: Non-alcoholic steatohepatitis
ER: Endoplasmic reticulum
UPR: Unfolded protein response
IRE1a: Inositol requiring enzyme 1a
PERK: Protein kinase (PKR)-like ER kinase
ATF6: Activating transcription factor 6
sXBP1: Spliced X-box-binding protein-1
CHOP: C/EBP homologous protein
ATF4: Activating transcription factor 4
TNF-α: Tumor necrosis factor-alpha
NF-κβ: Nuclear transcription factor-kappa B
VLDL: Very low-density lipoprotein
VLDLR: VLDL receptor
JNK: C-Jun N-terminal kinase
TLR: Toll-like receptor
PAMPs: Pathogen-associated molecular patterns
LPS: Lipopolysaccharide
DAMPs: Damage-associated molecular patterns
TGF-β1: Transforming growth factor-β1
hCLS: Hepatic crown-like structures
HFHC: High fat and high cholesterol
MCD: Methionine–choline deficient
HFD: High-fat diet
TRAIL: TNF-related apoptosis-inducing ligand
DC: Dendritic cells
MHC II: Major histocompatibility complex class II
HSCs: Hepatic stellate cells
BAFF: B cell activating factor
MyD88: Myeloid differentiation early response protein 88
